# Science vs Conspiracy: Collective Narratives in the Age of Misinformation

**DOI:** 10.1371/journal.pone.0118093

**Published:** 2015-02-23

**Authors:** Alessandro Bessi, Mauro Coletto, George Alexandru Davidescu, Antonio Scala, Guido Caldarelli, Walter Quattrociocchi

**Affiliations:** 1 IUSS Institute for Advanced Study, Pavia, Italy; 2 Laboratory of Computational Social Science, Networks Dept IMT Institute for Advanced Studies Lucca, Italy; 3 ISC-CNR Uos “Sapienza”, Roma, Italy; 4 Laboratory for the Modeling of Biological and Socio-technical Systems, Northeastern University, Boston, USA; University Toulouse 1 Capitole, FRANCE

## Abstract

The large availability of user provided contents on online social media facilitates people aggregation around shared beliefs, interests, worldviews and narratives. In spite of the enthusiastic rhetoric about the so called *collective intelligence* unsubstantiated rumors and conspiracy theories—e.g., chemtrails, reptilians or the Illuminati—are pervasive in online social networks (OSN). In this work we study, on a sample of 1.2 million of individuals, how information related to very distinct narratives—i.e. main stream scientific and conspiracy news—are consumed and shape communities on Facebook. Our results show that polarized communities emerge around distinct types of contents and usual consumers of conspiracy news result to be more focused and self-contained on their specific contents. To test potential biases induced by the continued exposure to unsubstantiated rumors on users’ content selection, we conclude our analysis measuring how users respond to 4,709 troll information—i.e. parodistic and sarcastic imitation of conspiracy theories. We find that 77.92% of likes and 80.86% of comments are from users usually interacting with conspiracy stories.

## Introduction

The World Wide Web has changed the dynamics of information transmission as well as the agenda-setting process [1]. Relevance of facts, in particular when related to social relevant issues, mingle with half-truths and untruths to create informational blends [2, 3]. In such a scenario, as pointed out by [4], individuals can be uninformed or misinformed and the role of corrections in the diffusion and formation of biased beliefs are not effective. In particular, in [5] online debunking campaigns have been shown to create a reinforcement effect in usual consumers of conspiracy stories. In this work, we address users consumption patterns of information using very distinct type of contents—i.e., main stream scientific news and conspiracy news. The former diffuse scientific knowledge and the sources are easy to access. The latter aim at diffusing what is neglected by *manipulated* main stream media. Specifically, conspiracy theses tend to reduce the complexity of reality by explaining significant social or political aspects as plots conceived by powerful individuals or organizations. Since these kinds of arguments can sometimes involve the rejection of science, alternative explanations are invoked to replace the scientific evidence. For instance, people who reject the link between HIV and AIDS generally believe that AIDS was created by the U.S. Government to control the African American population [6]. The spread of misinformation in such a context might be particularly difficult to detect and correct because of the social reinforcement—i.e. people are more likely to trust an information someway consistent with their system of beliefs [7–17]. The growth of knowledge fostered by an interconnected world together with the unprecedented acceleration of scientific progress has exposed the society to an increasing level of complexity to explain reality and its phenomena. Indeed, a shift of paradigm in the production and consumption of contents has occurred, utterly increasing the volumes as well as the heterogeneity of available to users. Everyone on the Web can produce, access and diffuse contents actively participating in the creation, diffusion and reinforcement of different narratives. Such a large heterogeneity of information fostered the aggregation of people around common interests, worldviews and narratives.

Narratives grounded on conspiracy theories tend to reduce the complexity of reality and are able to contain the uncertainty they generate [18–20]. They are able to create a climate of disengagement from mainstream society and from officially recommended practices [21]—e.g. vaccinations, diet, etc. Despite the enthusiastic rhetoric about the *collective intelligence* [22, 23] the role of socio-technical system in enforcing informed debates and their effects on the public opinion still remain unclear. However, the World Economic Forum listed massive digital misinformation as one of the main risks for modern society [24].

A multitude of mechanisms animates the flow and acceptance of false rumors, which in turn create false beliefs that are rarely corrected once adopted by an individual [8, 10, 25, 26]. The process of acceptance of a claim (whether documented or not) may be altered by normative social influence or by the coherence with the system of beliefs if the individual [27, 28]. A large body of literature addresses the study of social dynamics on socio-technical systems from social contagion up to social reinforcement [12–15, 17, 29–41].

Recently in [42, 43] it has been shown that online unsubstantiated rumors—such as the link between vaccines and autism, the global warming induced by chem-trails or the secret alien government—and main stream information—such as scientific news and updates—reverberate in a comparable way. Pervasiveness of unreliable contents might lead to mix up unsubstantiated stories with their satirical counterparts—e.g. the presence of sildenafil-citratum (the active ingredient of Viagra™) [44] in chem-trails or the anti hypnotic effects of lemons (more than 45000 shares on Facebook) [45, 46]. In fact, there are very distinct groups, namely *trolls*, building Facebook pages as a caricatural version of conspiracy news. Their activities range from controversial comments and posting satirical contents mimicking conspiracy news sources, to the fabrication of purely fictitious statements, heavily unrealistic and sarcastic. Not rarely, these memes became viral and were used as evidence in online debates from political activists [47].

In this work we target consumption patterns of users with respect to very distinct types of information. Focusing on the Italian context and helped by pages very active in debunking unsubstantiated rumors (see acknowledgment section), we build an atlas of scientific and conspiracy information sources on Facebook. Our dataset contains 271,296 post created by 73 Facebook pages. Pages are classified according to the kind of information disseminated and their self description in conspiracy news—alternative explanations of reality aiming at diffusing contents neglected by main stream information—and scientific news. For further details about the data collection and the dataset refer to the Methods section. Notice that it is not our intention claiming that conspiracy information are necessarily false. Our focus is on how communities formed around different information and narratives interact and consume their preferred information.

In the analysis, we account for user interaction with respect to pages public posts—i.e. likes, shares, and comments. Each of these actions has a particular meaning [48–50]. A *like* stands for a positive feedback to the post; a *share* expresses the will to increase the visibility of a given information; and *comment* is the way in which online collective debates take form around the topic promoted by posts. Comments may contain negative or positive feedbacks with respect to the post. Our analysis starts with an outline of information consumption patterns and the community structure of pages according to their common users. We label polarized users—users which their like activity (positive feedback) is almost (95%) exclusively on the pages of one category—and find similar interaction patterns on the two communities with respect to preferred contents. According to literature on opinion dynamics [37], in particular the one related to the Bounded confidence model (BCM) [51]—two individuals are able to influence each other only if the distance between their opinion is below a given distance—users consuming different and opposite information tend to aggregate into isolated clusters (*polarization*). Moreover, we measure their commenting activity on the opposite category finding that polarized users of conspiracy news are more focused on posts of their community and that they are more oriented on the diffusion of their contents—i.e. they are more prone to like and share posts from conspiracy pages. On the other hand, usual consumers of scientific news result to be less committed in the diffusion and more prone to comment on conspiracy pages. Finally, we test the response of polarized users to the exposure to 4709 satirical and demential version of conspiracy stories finding that, out of 3888 users labeled on likes and 3959 on comments, the most of them are usual consumers of conspiracy stories (80.86% of likes and 77.92% of comments). Our findings, coherently with [52–54] indicate that the relationship between beliefs in conspiracy theories and the need for cognitive closure—i.e. the attitude of conspiracists to avoid profound scrutiny of evidence to a given matter of fact—is the driving factors for the diffusion of false claims.

## Results and discussion

In this work we address the driving forces behind the popularity of contents on online social media To do this, we start our analysis by characterizing users’ interaction patterns with respect to different kind of contents. Then, we label typical users according to the kind of information they are usually exposed to and validate their tolerance with respect to information that we know to be false as they are a parodistic imitation of conspiracy stories containing fictitious and heavily unrealistic statements.

### Consumption patterns on science and conspiracy news

**Fig 1:**
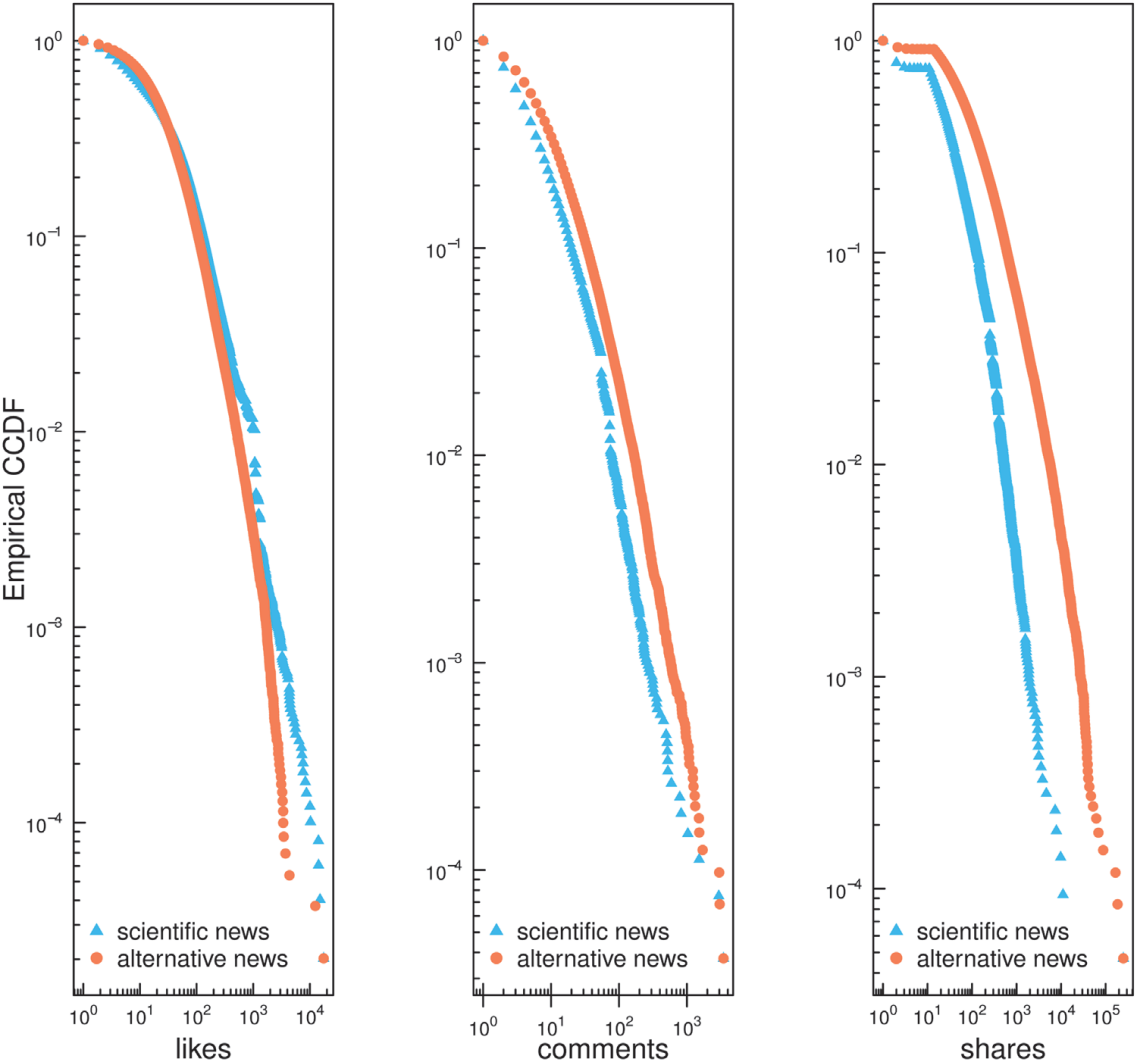
Users Activity. Empirical complementary cumulative distribution function (CCDF) of users’ activity (like, comment and share) for post grouped by page category. The distributions are indicating heavy–tailed consumption patterns for the various pages.

Our analysis starts by looking at how Facebook users interact with contents from pages of conspiracy and mainstream scientific news. Fig. 1 shows the empirical complementary cumulative distribution function (CCDF) for likes (intended as positive feedbacks to the post), comments (a measure of the activity of online collective debates), and shares (intended as the the will to increase the visibility of a given information) for all posts produced by the different categories of pages. Distributions of likes, comments, and shares on both categories are heavy–tailed.

**Fig 2:**
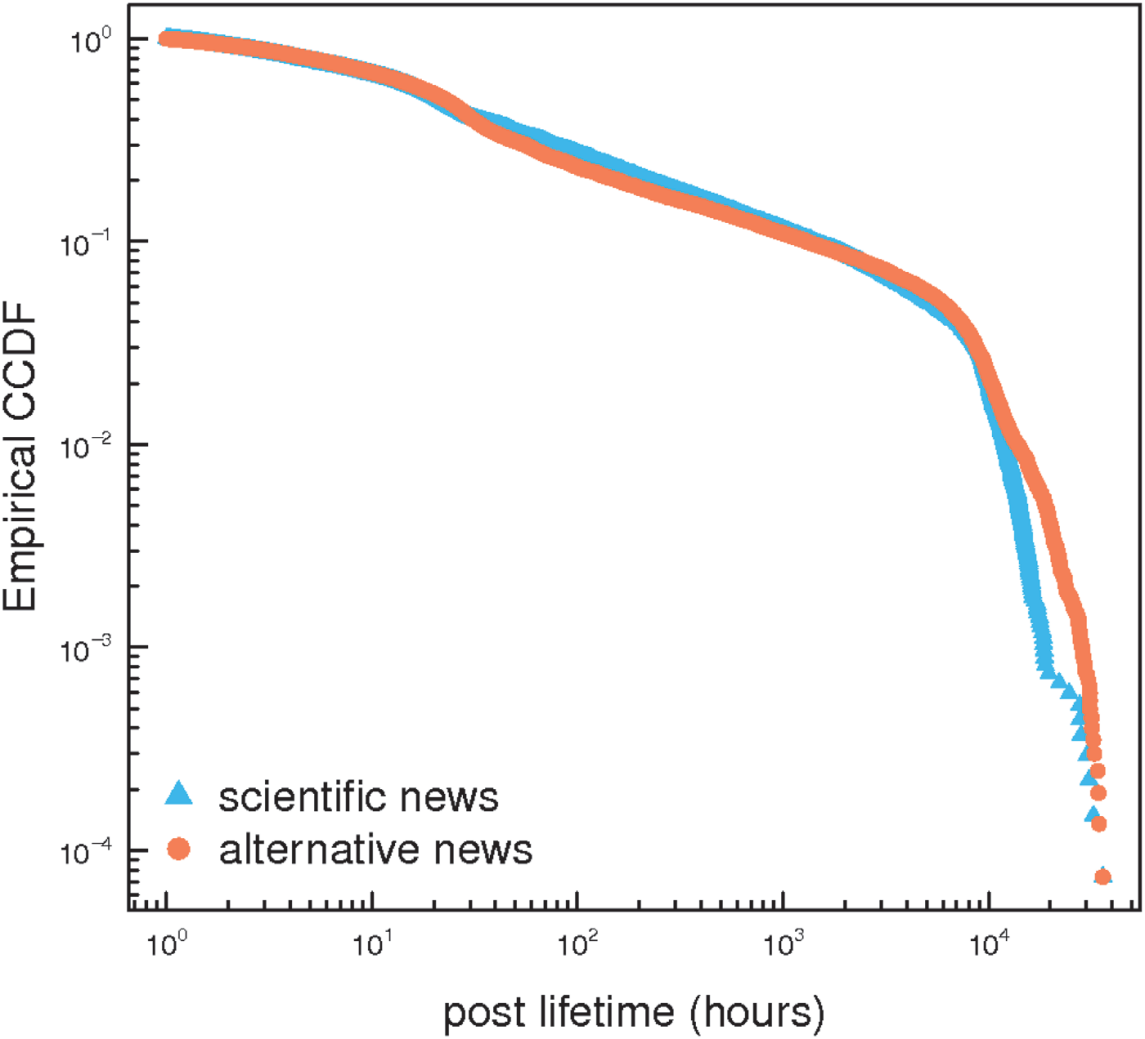
Post lifetime. Empirical complementary cumulative distribution function (CCDF), grouped by page category, of the temporal distance between the first and last comment to each post. The life time of posts in both categories is similar.

A post sets the attention on a given topic, then a discussion may evolve in the form of comments. To further investigate users consumption patterns, we zoom in at the level of comments. Such a measure is a good approximation of users attention with respect to the information reported on by the post. In Fig. 2 we show CCDF of the posts lifetime—i.e. the temporal distance between the first and the last comment for each post from the two categories of pages. Very distinct kinds of contents have have a comparable lifetime.

To account for the distinctive features of the consumption patterns related to different contents, we focus on the correlation of combination of users’ interactions with posts. Likes and comments have a different meaning from a user viewpoint. Most of the time, a like stands for a positive feedback to the post; a share expresses the will to increase the visibility of a given information; and a comment is the way in which online collective debates take form and may contain negative or positive feedbacks with respect to the post. Notice that, cases in which they are motivated by ironic reasons are impossible to detect. In order to compute the correlation among different actions, we use the Pearson coefficient—i.e., the covariance of two variables (in this case couples of action) divided by the product of their standard deviations. In Table 1 we show the Pearson correlation for user couple of actions on posts (likes, comments and shares). As an example, a high correlation coefficient for Comments/Shares indicates that posts more commented are likely to be shared and vice versa.

**Table 1:** Users Actions. Correlation (Pearson coefficient) between couple of actions to each post in scientific and conspiracy news. Posts from conspiracy pages are more likely to be liked and shared by users, indicating a major commitment in the diffusion.

	**Likes/Comments**	**Likes/Shares**	**Comments/Shares**
Science	0.523	0.218	0.522
Conspiracy	0.639	**0.816**	0.658

Correlation values for posts of conspiracy news have higher values than those in science news. They receive more likes and shares, indicating a preference of conspiracy users to promote their liked contents. This finding is consistent with [52–54] which state that conspiracists need for cognitive closure, i.e. they are more likely to interact with conspiracy based theories and have a lower trust in other information sources. Qualitatively different information are consumed in a comparable way. However, zooming in at the combination of actions we find that users of conspiracy pages are more prone to share and like on a post. Such a latter result indicates a higher level of commitment of consumers of conspiracy news. They are more oriented to the diffusion of conspiracy related topics that are—according to their system of beliefs—neglected by main stream media and scientific news and consequently very difficult to verify. Such a pattern oriented to diffusion of conspiracy news opens to interesting about the pervasiveness of unsubstantiated rumors in online social media.

### Information-based communities

**Fig 3:**
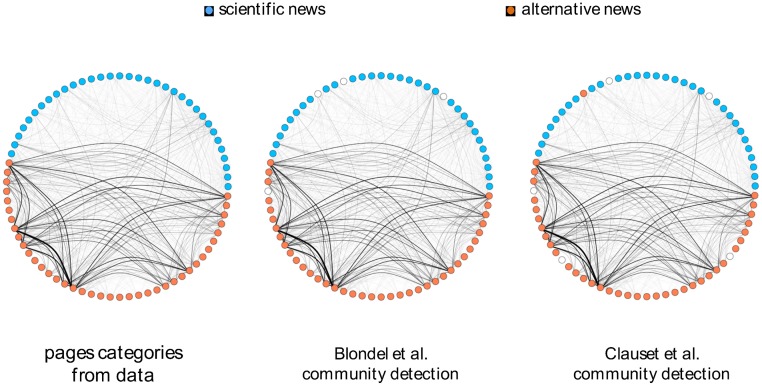
Page Network. The membership of 73 pages as a) identified by means of their self-description, b) by applying the multi-level modularity optimization algorithm, and c) by looking at the maximum modularity score. Community detection algorithms based on modularity are good discriminants for community partitioning.

The classification of pages in science and conspiracy related contents is grounded on their self-description and on the kind of promoted content (see the Methods section for further details and the list of pages). We want to understand if users engagement across very distinct contents shapes different communities around contents. We apply a network based approach aimed at measuring distinctive connectivity patterns of these information-based communities? i.e., users consuming information belonging to the same narrative. In particular, we transform data in order to have a bipartite network of pages and users—i.e., two pages are connected if a user liked a post from both of them. In Fig. 3 we show the membership of pages (orange for conspiracy and azure for science). In the first panel, memberships are given according to our categorization of pages (for further details refer to the Methods section). The second panel shows the page network with membership given by applying the multi-level modularity optimization algorithm [55]. In the third panel, membership is obtained by applying an algorithm that looks for the maximum modularity score [56].

These findings indicates that connectivity patterns, in particular the modularity, between the two categories of pages differ. Since we are considering users’ likes on the pages’ posts, this aspect is pointing out a higher mobility of users of across pages of the conspiracy category.

### Polarized users and their interaction patterns

In this section we focus on the users engagement across the different contents. Hence, we label users by means of a simple thresholding algorithm accounting for the percentage of likes on one or the other category. Notice that the choice of the *like* as a discriminant is grounded on the fact that generally such an action stands for a positive feedback to a post [50]. We consider a user to be polarized in a community when the number of his/her likes with respect to his/her total like activity on one category—scientific or conspiracy news—is higher than 95% (for further details about the algorithm refer to the Methods section). We identify 255,225 polarized users of scientific pages—i.e., resulting t be the 76,79% of users interacted on scientific pages) and 790,899 conspiracy polarized users—i.e., the 91,53% of users interacting with conspiracy pages in terms of liking. Users activity across pages is highly polarized. According to literature on opinion dynamics [37] in particular the one related to the Bounded Confidence Model (BCM) [51]—two nodes are able to influence each other only if the distance between their opinions is below a given distance—users consuming different and opposite information tend to form polarized clusters. The same hold If we look at commenting activity of polarized users inside and outside their community. In particular, those users that are polarized on conspiracy news tend to interact especially in their community both in terms of comments (99,08%) and likes. Users polarized in science tend to comment slightly more outside their community (90,29%). Results are summarized in Table 2.

**Table 2:** Activity of polarized users. Number of classified users for each category and their commenting activity on the category in which they are classified and on the opposite category. Users polarized on conspiracy pages tend to interact especially in their community both in terms of comments and likes. Users polarized in science are more active elsewhere.

	**Users classified**	**(%) Users classified**	**Comments on their category**	**Comments on the opposite category**	**Comments on both categories**
Science News	255,225	76,79	126,454	13,603	140,057
Conspiracy News	790,899	91,53	642,229	5,954	648,183

**Fig 4:**
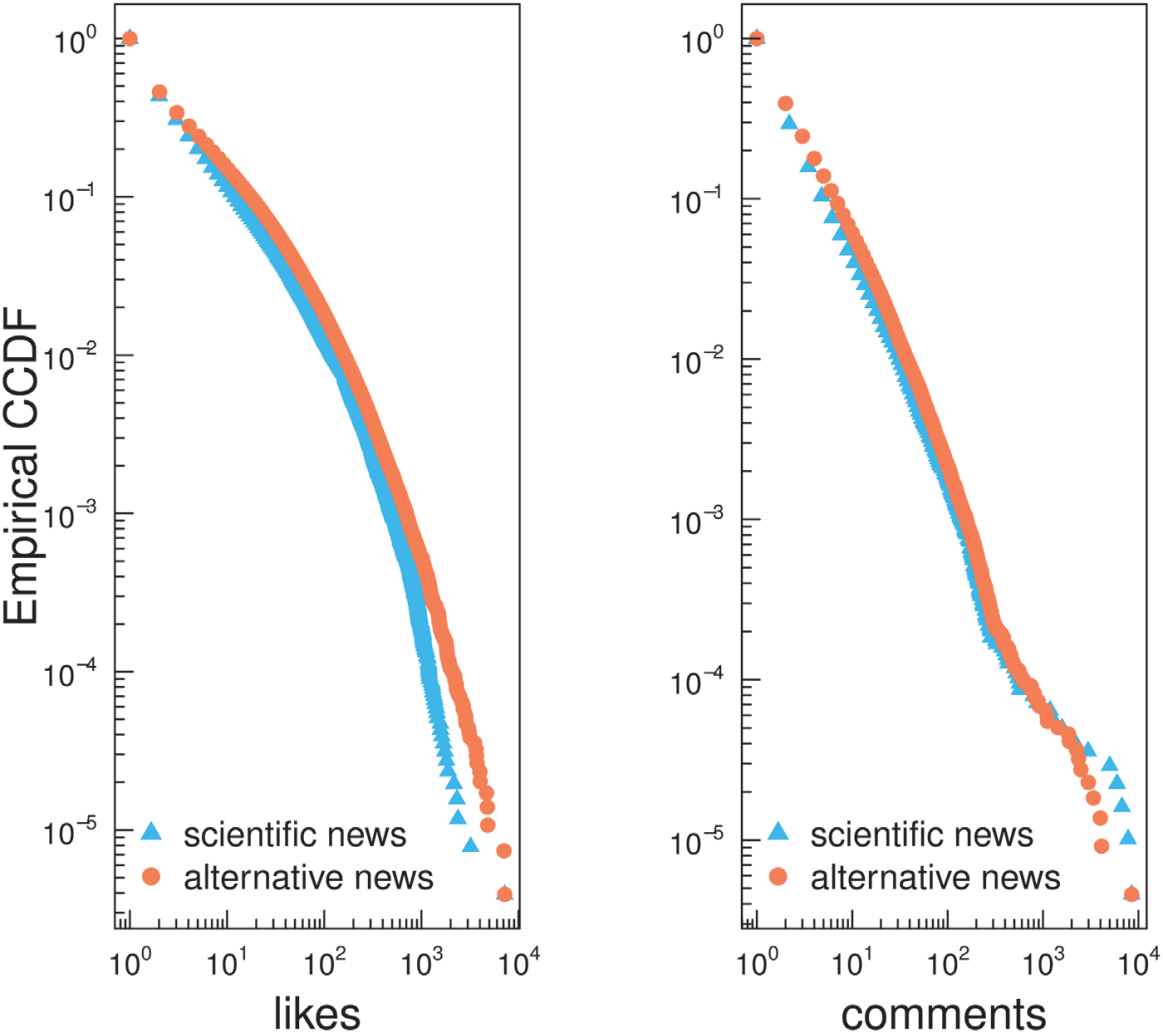
Consumption patterns of polarized users. Empirical complementary cumulative distribution function (CCDF) for likes and comments of polarized users.

Fig. 4 shows the CCDF for likes and comments of polarized users. Despite the very profound different nature of contents, consumption patterns are nearly the same both in terms of likes and comments. This finding indicates that very engaged users of different and clustered communities formed around different kind of narratives consume their preferred information in a similar way.

**Fig 5:**
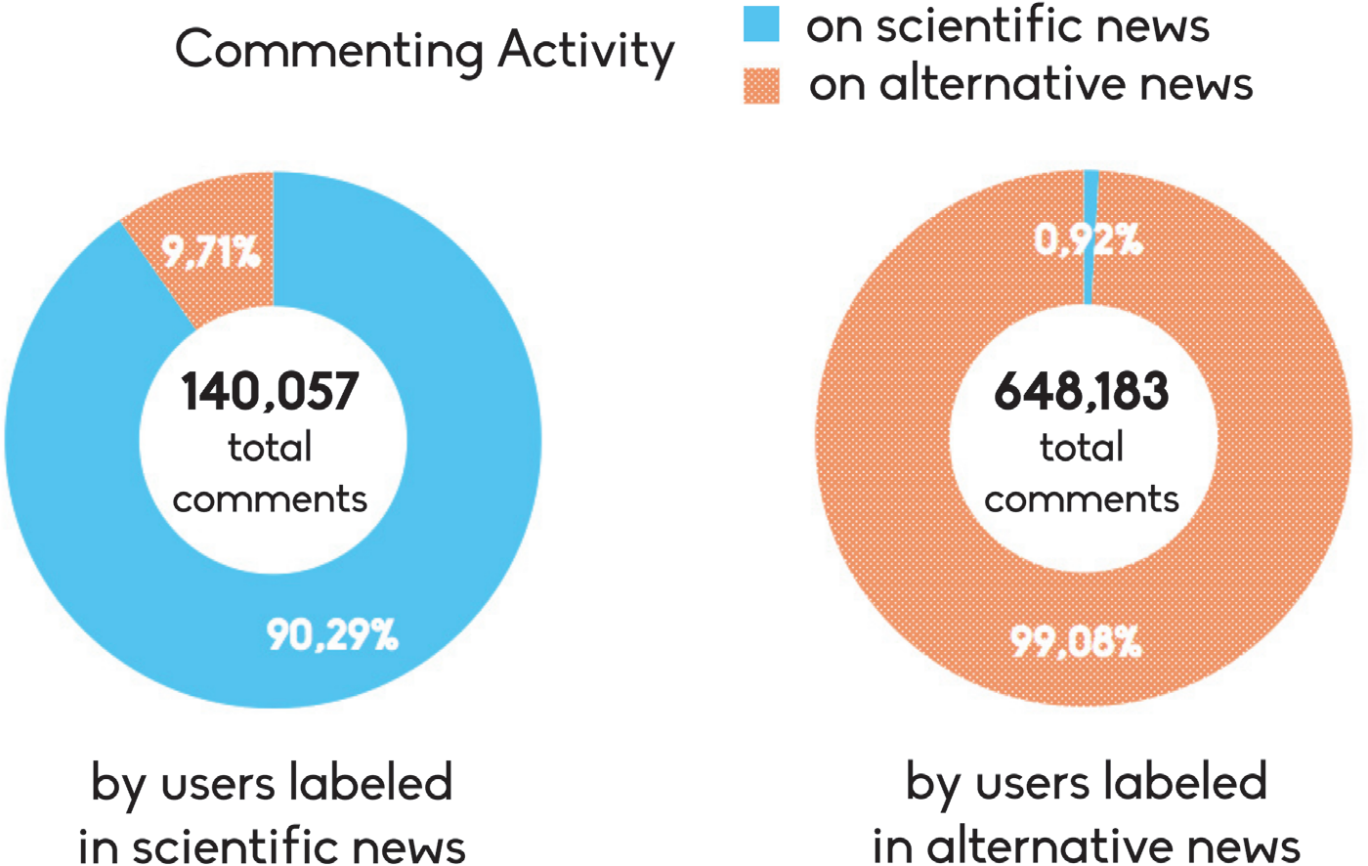
Activity and communities. Posts on which at least a member of each the two communities has commented. The number of posts is 7,751 (1,991 from scientific news and 5,760 from conspiracy news). Here we show the commenting activity in terms of polarized users on the two categories.

As a further investigation, we focus on the post where polarized users of both communities commented. Hence, we select the set of posts on which at least a polarized user of each of the two communities has commented. We find polarized users of communities debating on 7,751 posts (1,991 from science news and 5,760 from conspiracy news). The post at the interface, where the two communities discuss are mainly on the conspiracy side. As shown in Fig. 5, polarized users of scientific news made 13,603 comments on post published by conspiracy news (9.71% of their total commenting activity), whereas polarized users of conspiracy news commented on scientific posts only 5,954 times (0.92% of their total commenting activity, i.e. roughly ten times less than polarized users of scientific news).

### Response to false information

On online social networks, users discover and share information with their friends and through *cascades* of reshares information might reach a large number of individuals. Interesting is the popular case of Senator Cirenga’s [57, 58] law proposing to fund policy makers with 134 billion of euros (10% of the Italian GDP) in case of defeat in the political competition. This was an intentional joke with an explicit mention to its satirical nature. The case of Senator Cirenga became popular within online political activists and used as an argumentation in political debates [47].

Our analysis showed that users tend to aggregate around preferred contents shaping well defined groups having similar information consumption patterns. Our hypothesis is that the exposure to unsubstantiated claims (that are pervasive in online social media) might affect user selection criteria by increasing the attitude to interact with false information. Therefore, in this section we want to test how polarized users usually exposed to distinct narrative—one that can be veriefied (science news) and one that by definition is almost impossible to check—interact with posts that are deliberately false.

**Fig 6:**
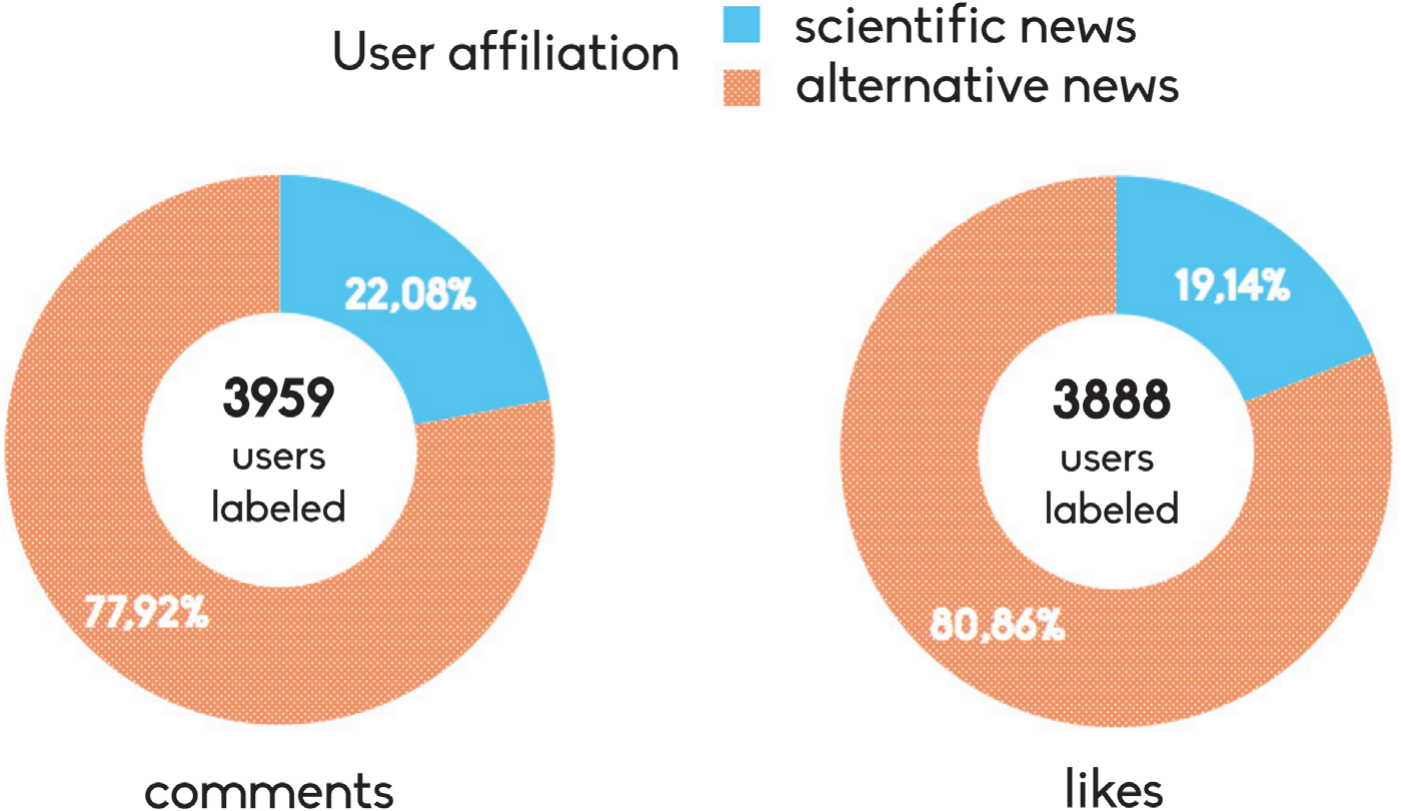
Polarized users on false information. Percentage of comments and likes on intentional false memes posted by a satirical page from polarized users of the two categories.

To do this we collected a set of troll posts—i.e. paradoxical imitations of conspiracy information sources. These posts are clearly unsubstantiated claims, like the undisclosed news that infinite energy has been finally discovered, or that a new lamp made of actinides (e.g. plutonium and uranium) might solve problems of energy gathering with less impact on the environment, or that the chemical analysis revealed that chem-trails contains sildenafil citratum (the active ingredient of Viagra™). Fig. 6 shows how polarized users of both categories interact with troll posts in terms of comments and likes. We find that polarized users of conspiracy pages are more active in liking and commenting on intentionally false claims.

## Conclusions

Recently in [42, 43] has been shown that unsubstantiated claims reverberate for a timespan comparable to the one of more verified information and that usual consumers of conspiracy theories are more prone to interact with them. Conspiracy theories find on the internet a natural medium for their diffusion and, not rarely, trigger collective counter-conspirational actions [59, 60]. Narratives grounded on conspiracy theories tend to reduce the complexity of reality and are able to contain the uncertainty they generate [18–20]. In this work we studied how users interact with information related to different (opposite) narratives on Facebook. Through a thresholding algorithm we label polarized users on the two categories of pages identifying well shaped communities. In particular, we measure commenting activity of polarized users on the opposite category, finding that polarized users of conspiracy news are more focused on posts of their community and their attention is more oriented to diffuse conspiracy contents. On the other hand, polarized users of scientific news are less committed in the diffusion and more prone to comment on conspiracy pages. A possible explanation for such a behavior is that the former want to diffuse what is neglected by main stream thinking, whereas the latter aims at inhibiting the diffusion of conspiracy news and proliferation of narratives based on unsubstantiated claims. Finally, we test how polarized users of both categories responded to the inoculation of 4,709 false claims produced by a parodistic page, finding polarized users of conspiracy pages to be the most active.

These results are coherent with the findings of [52–54] indicating the existence of a relationship between beliefs in conspiracy theories and the need for cognitive closure. Those who use a more heuristic approach when evaluating evidences to form their opinions are more likely to end up with an account more consistent with their existing system of beliefs. However, anti-conspiracy theorists may not only reject evidence that points toward a conspiracy theory account, but also spend cognitive resources for seeking out evidences to debunk conspiracy theories even when these are satirical imitation of false claims. These results open to new possibilities to understand popularity of information in online social media beyond simple structural metrics. Furthermore, we show that where unsubstantiated rumors are pervasive, false rumors might easy proliferate. Next envisioned steps for our research is to look at reactions of users to different kind of information according to a more detailed classification on contents.

## Methods

### Ethics Statement

The entire data collection process has been carried out exclusively through the Facebook Graph API [61], which is publicly available, and for the analysis (according to the specification settings of the API) we used only public available data (users with privacy restrictions are not included in the dataset). The pages from which we download data are public Facebook entities (can be accessed by anyone). User content contributing to such pages is also public unless the user’s privacy settings specify otherwise and in that case it is not available to us.

### Data collection

In this study we address the effect of the usual exposure to diverse verifiable contents on the diffusion of false rumors. We identified two main categories of pages: conspiracy news—i.e. pages promoting contents *neglected* by main stream media—and science news. We defined the space of our investigation with the help of Facebook groups very active in debunking conspiracy theses (*Protesi di Protesi di Complotto*, *Che vuol dire reale*, *La menzogna diventa verita e passa alla storia*). We categorized page according to their contents and their self description.

Concerning conspiracy news, their self description is often claiming the mission to inform people about topics neglected by main stream media. Pages like *Scienza di Confine*, *Lo Sai* or *CoscienzaSveglia* promote heterogeneous contents ranging from aliens, chemtrails, geocentrism, up to the causal relation between vaccinations and homosexuality. We do not focus on the truth value of their information but rather on the possibility to verify their claims. Conversely, science news—e.g *Scientificast*, *Italia unita per la scienza* are active in diffusing posts about the most recent scientific advances. The selection of the source has been iterated several times and verified by all the authors. To our knowledge, the final dataset is the complete set of all scientific and conspiracist information sources active in the Italian Facebook scenario. In addition, we identify two pages posting satirical news with the aim of mocking usual rumors circulating on line by adding satirical contents.

The pages from which we downloaded data are public Facebook entities (can be accessed by virtually anyone). The resulting dataset is composed of 73 public pages divided in scientific and conspiracist news for which we downloaded all the posts (and their respective users interactions) over a timespan of 4 years (2010 to 2014).

The exact breakdown of the data is presented in Table 3. The first category includes all pages diffusing conspiracy information—pages which disseminate controversial information, most often lacking supporting evidence and sometimes contradictory of the official news (i.e. conspiracy theories). The second category is that of scientific dissemination including scientific institutions and scientific press having the main mission to diffuse scientific knowledge.

**Table 3:** Breakdown of Facebook dataset. The number of pages, posts, likes, comments, likers, and commenters for conspiracy and science news.

	**Total**	**Science News**	**Conspiracy News**
Pages	73	34	39
Posts	271,296	62,705	208,591
Likes	9,164,781	2,505,399	6,659,382
Comments	1,017,509	180,918	836,591
Likers	1,196,404	332,357	864,047
Commenters	279,972	53,438	226,534

### Preliminaries and Definitions

**Statistical Tools**. To characterize random variables, a main tool is the probability distribution function (PDF), which gives the probability that a random variable *X* assumes a value in the interval [*a*, *b*], i.e. $P(a\leq X\leq b)=\int_{a}^{b}f(x)dx$. The cumulative distribution function (CDF) is another important tool giving the probability that a random variable *X* is less than or equal to a given value *x*, i.e. $F(x)=P(X\leq x)=\int_{-\infty }^{x}f(y)dy$. In social sciences, an often occuring probability distribution function is the Pareto’s law *f*(*x*) ∼ *x* ^−*γ*^, that is characterized by power law tails, i.e. by the occurrence of rare but relevant events. In fact, while *f*(*x*) → 0 for *x* → ∞ (i.e. high values of a random variable *X* are rare), the total probability of rare events is given by $C(x)=P(X>x)=\int_{x}^{\infty }f(y)dy$, where *x* is a sufficiently large value. Notice that *C*(*x*) is the Complement to the CDF (CCDF), where complement indicates that *C*(*x*) = 1 − *F*(*x*). Hence, in order to better visualize the behavior of empirical heavy–tailed distributions, we recur to log–log plots of the CCDF.

**Bipartite Networks and Community Detection**. We consider a bipartite network having as nodes users and affiliation the Facebook pages. A comment to a given information posted by a page determines a link between a user and a page. More formally, a bipartite graph is a triple G=(A,B,E) where *A* = {*a*_*i*_ ∣ *i* = 1 … *n*_*A*_} and *B* = {*b*_*j*_ ∣ *j* = 1 … *n*_*B*_} are two disjoint sets of vertices, and *E* ⊆ *A* × *B* is the set of edges—i.e. edges exist only between vertices of the two different sets *A* and *B*. The bipartite graph G is described by the matrix *M* defined as

$$\begin{array}{c} M_{ij}=\left\{\begin{array}{cc} 1 & if\;an\;edge\;exists\;between\;a_{i}\;and\;b_{j}\\ 0 & otherwise \end{array}\right. \end{array}$$

For our analysis we use the co-occurrence matrices *C* ^*A*^ = *MM* ^*T*^ and *C* ^*B*^ = *M* ^*T*^ *M* that count, respectively, the number of common neighbors between two vertices of *A* or *B*. *C* ^*A*^ is the weighted adjacency matrix of the co-occurrence graph $C^{A}$ with vertices on *A*. Each non-zero element of *C* ^*A*^ corresponds to an edge among vertices *a*_*i*_ and *a*_*j*_ with weight $P_{ij}^{A}$. To test the community partitioning we use two well known community detection algorithms based on modularity [55, 56]. The former algorithm is based on multi-level modularity optimization. Initially, each vertex is assigned to a community on its own. In every step, vertices are re-assigned to communities in a local, greedy way. Nodes are moved to the community in which they achieve the highest modularity. Differently, the latter algorithm looks for the maximum modularity score by considering all possible community structures in the network. We apply both algorithms to the bipartite projection on pages.

**Fig 7:**
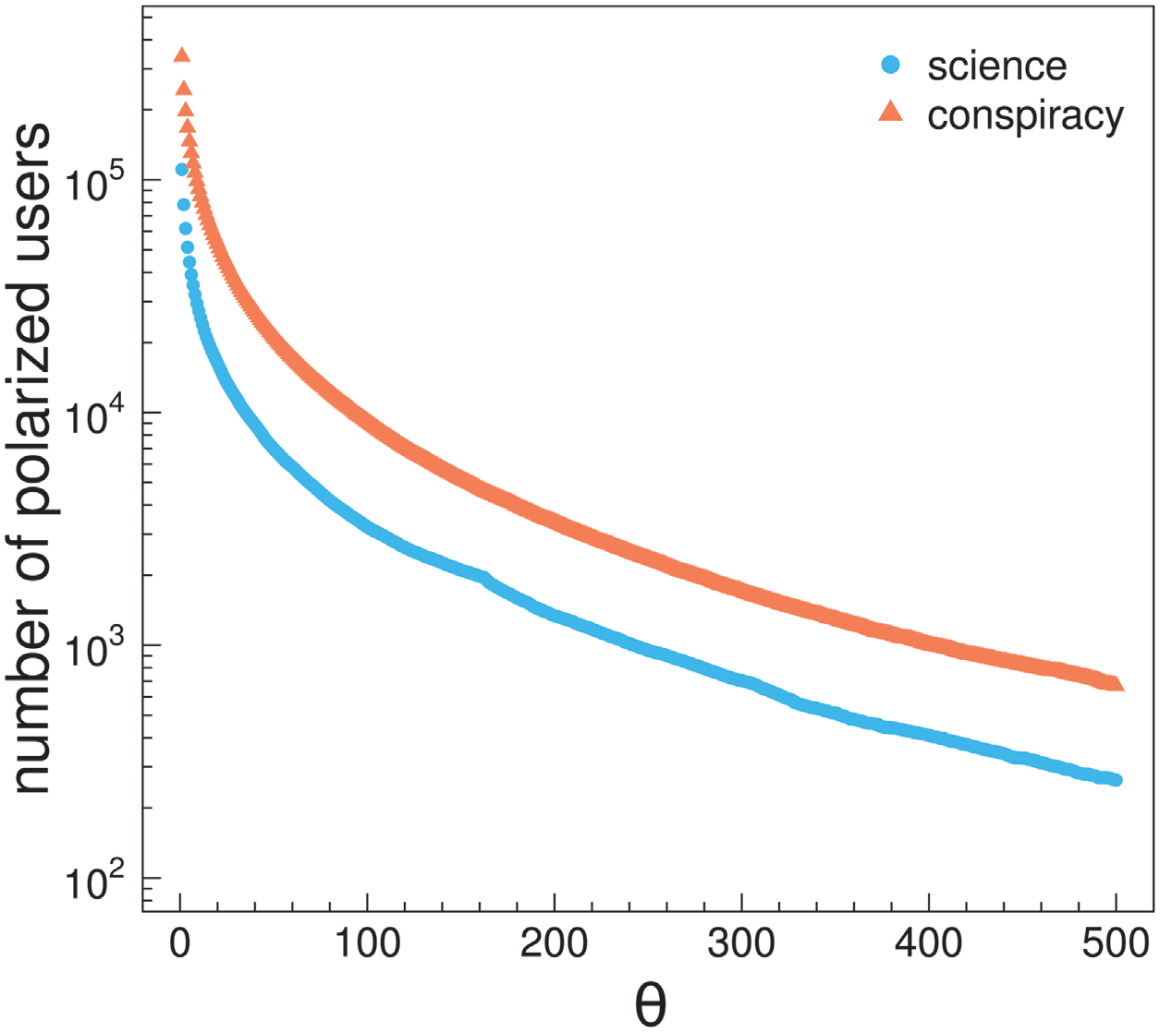
Polarized users and activity. Number of polarized users as a function of the thresholding value *θ* on the two categories.

**Labeling algorithm**. The labeling algorithm can be described as thresholding strategy on the total number of users likes. Considering the total number of likes of a user *L*_*u*_ on both posts *P* in categories *S* and *C*. Let *l*_*s*_ and *l*_*c*_ define the number of likes of a user *u* on *P*_*s*_ or *P*_*c*_, respectively denoting posts from scientific and conspiracy pages. Then, we will have the total like activity of users on one category expressed as $\frac{l_{s}}{L_{u}}$. Fixing a threshold *θ* we can discriminate users with enough activity on one category. More precisely, the condition for a user to be labeled as a polarized user in one category can be described as $\frac{l_{s}}{L_{u}}$ ∨ $\frac{l_{c}}{L_{u}}>\theta$. In Fig. 7 we show the number of polarized users as a function of *θ*. Both curves decrease with a comparable rate.

### List of pages

In this section are listed pages of our dataset. In Table 4 the list of scientific news and on Table 5 the list of conspiracy pages.

**Table 4:** Scientific news sources. List of Facebook pages diffusing main stream scientific news and their url.

	**Page Name**	**Link**
1	Scientificast.it	www.facebook.com/129133110517884
2	CICAP	www.facebook.com/32775139194
3	OggiScienza	www.facebook.com/106965734432
4	Query	www.facebook.com/128523133833337
5	Gravit Zero	www.facebook.com/138484279514358
6	COELUM Astronomia	www.facebook.com/81631306737
7	MedBunker	www.facebook.com/246240278737917
8	In Difesa della Sperimentazione Animale	www.facebook.com/365212740272738
9	Italia Unita per la Scienza	www.facebook.com/492924810790346
10	Scienza Live	www.facebook.com/227175397415634
11	La scienza come non l’avete mai vista	www.facebook.com/230542647135219
12	LIBERASCIENZA	www.facebook.com/301266998787
13	Scienze Naturali	www.facebook.com/134760945225
14	Perch vaccino	www.facebook.com/338627506257240
15	Le Scienze	www.facebook.com/146489812096483
16	Vera scienza	www.facebook.com/389493082245
17	Scienza in rete	www.facebook.com/84645527341
18	Galileo, giornale di scienza e problemi globali	www.facebook.com/94897729756
19	Scie Chimiche: Informazione Corretta	www.facebook.com/351626174626
20	Complottismo? No grazie	www.facebook.com/399888818975
21	INFN—Istituto Nazionale di Fisica Nucleare	www.facebook.com/45086217578
22	Signoraggio: informazione corretta	www.facebook.com/279217954594
23	JFK informazione corretta	www.facebook.com/113204388784459
24	Scetticamente	www.facebook.com/146529622080908
25	Vivisezione e Sperimentazione Animale, verit e menzogne	www.facebook.com/548684548518541
26	Medici Senza Frontiere	www.facebook.com/65737832194
27	Task Force Pandora	www.facebook.com/273189619499850
28	VaccinarSI	www.facebook.com/148150648573922
29	Lega Nerd	www.facebook.com/165086498710
30	Super Quark	www.facebook.com/47601641660
31	Curiosit Scientifiche	www.facebook.com/595492993822831
32	Minerva—Associazione di Divulgazione Scientifica	www.facebook.com/161460900714958
33	Pro-Test Italia	www.facebook.com/221292424664911
34	Uniti per la Ricerca	www.facebook.com/132734716745038

**Table 5:** Conspiracy news sources. List of Facebook pages diffusing conspiracy news and their url.

	**Page Name**	**Link**
1	Scienza di Confine	www.facebook.com/188189217954979
2	CSSC—Cieli Senza Scie Chimiche	www.facebook.com/253520844711659
3	STOP ALLE SCIE CHIMICHE	www.facebook.com/199277020680
4	Vaccini Basta	www.facebook.com/233426770069342
5	Tanker Enemy	www.facebook.com/444154468988487
6	SCIE CHIMICHE	www.facebook.com/68091825232
7	MES Dittatore Europeo	www.facebook.com/194120424046954
8	Lo sai	www.facebook.com/126393880733870
9	AmbienteBio	www.facebook.com/109383485816534
10	Eco(R)esistenza	www.facebook.com/203737476337348
11	curarsialnaturale	www.facebook.com/159590407439801
12	La Resistenza	www.facebook.com/256612957830788
13	Radical Bio	www.facebook.com/124489267724876
14	Fuori da Matrix	www.facebook.com/123944574364433
15	Graviola Italia	www.facebook.com/130541730433071
16	Signoraggio.it	www.facebook.com/278440415537619
17	Informare Per Resistere	www.facebook.com/101748583911
18	Sul Nuovo Ordine Mondiale	www.facebook.com/340262489362734
19	Avvistamenti e Contatti	www.facebook.com/352513104826417
20	Umani in Divenire	www.facebook.com/195235103879949
21	Nikola Tesla—il SEGRETO	www.facebook.com/108255081924
22	Teletrasporto	www.facebook.com/100774912863
23	PNL e Ipnosi	www.facebook.com/150500394993159
24	HAARP—controllo climatico	www.facebook.com/117166361628599
25	Sezione Aurea, Studio di Energia Vibrazionale	www.facebook.com/113640815379825
26	PER UNA NUOVA MEDICINA	www.facebook.com/113933508706361
27	PSICOALIMENTARSI E CURARSI NATURALMENTE	www.facebook.com/119866258041409
28	La nostra ignoranza la LORO forza.	www.facebook.com/520400687983468
29	HIV non causa AIDS	www.facebook.com/121365461259470
30	Sapere un Dovere	www.facebook.com/444729718909881
31	V per Verit	www.facebook.com/223425924337104
32	Genitori veg	www.facebook.com/211328765641743
33	Operatori di luce	www.facebook.com/195636673927835
34	Coscienza Nuova	www.facebook.com/292747470828855
35	Aprite Gli Occhi	www.facebook.com/145389958854351
36	Neovitruvian	www.facebook.com/128660840526907
37	CoscienzaSveglia	www.facebook.com/158362357555710
38	Medicinenon	www.facebook.com/248246118546060
39	TERRA REAL TIME	www.facebook.com/208776375809817
